# Circulating Interleukin-37 as a Biomarker Candidate for Hepatocellular Carcinoma in Decompensated Advanced Chronic Liver Disease

**DOI:** 10.3390/life16040563

**Published:** 2026-03-30

**Authors:** Michael Mederer, Johanna Piater, Felix Keller, Barbara Enrich, Veronika Cibulkova, Valeria Wagner, Rebecca Giquel-Fernandes, Andreas Kronbichler, Herbert Tilg, Maria Effenberger

**Affiliations:** 1 Department of Internal Medicine I, Gastroenterology, Hepatology, Endocrinology & Metabolism, Medical University of Innsbruck, 6020 Innsbruck, Austria; 2 Department of Internal Medicine IV, Nephrology & Hypertension, Medical University of Innsbruck, 6020 Innsbruck, Austria

**Keywords:** HCC, IL-37, dACLD, cACLD, ACLD, biomarker, alpha-fetoprotein, cirrhosis

## Abstract

Hepatocellular carcinoma (HCC) remains a leading cause of mortality in patients with advanced chronic liver disease (ACLD), particularly in those with decompensated cirrhosis, where traditional biomarkers such as alpha-fetoprotein (AFP) often fail to reliably detect malignancy. Interleukin-37 (IL-37), an anti-inflammatory cytokine with reported tumour-suppressive properties, has emerged as a candidate biomarker in hepatocarcinogenesis. This prospective study investigated serum IL-37 concentrations in 221 patients with ACLD (54 with HCC and 167 without HCC). IL-37 was measured at the time of clinical assessment, and routine laboratory parameters, disease severity scores (MELD, Child–Pugh), and tumour staging (BCLC, LI-RADS) were recorded. IL-37 levels were not significantly different in patients with compensated ACLD (cACLD) with or without HCC. In contrast, in decompensated ACLD (dACLD), IL-37 concentrations were significantly lower in patients with HCC, particularly in those with advanced hepatic dysfunction. Stratified analyses revealed an inverse relationship between IL-37 and AFP in cACLD, whereas in dACLD, IL-37 appeared more informative, as AFP levels were affected by systemic inflammation. Patients with prevalent HCC exhibited numerically lower IL-37 compared with those who developed HCC during follow-up, suggesting that IL-37 decline may precede overt tumour manifestation. Kaplan–Meier survival analysis showed a trend toward improved overall survival in patients with higher IL-37 levels, although this did not reach statistical significance. These findings highlight IL-37 as a promising biomarker candidate that might reflect immune regulation and tumour biology in ACLD. In particular, IL-37 may complement AFP for HCC detection in decompensated cirrhosis, where conventional biomarkers often fail. Future studies with larger, longitudinal cohorts are warranted to validate IL-37 as a predictive and prognostic marker in high-risk populations.

## 1. Introduction

Hepatocellular carcinoma (HCC) is the most prevalent primary liver malignancy and a major contributor to global cancer mortality [1,2]. Most cases arise on a background of chronic liver disease and cirrhosis, where sustained inflammation promotes hepatocyte injury, fibrogenesis, and malignant transformation [2,3,4,5]. Chronic liver injury related to viral hepatitis, alcohol, and metabolic dysfunction triggers immune responses and cytokine cascades that not only drive fibrosis but also create a tumour-promoting microenvironment, linking inflammation to carcinogenesis [3,4,5,6]. Inflammatory cytokines and signalling pathways in chronic liver disease and cirrhosis have been well described, with cytokine dysregulation implicated in hepatocarcinogenesis and progression of liver pathology [3,4,5,6,7]. In this context, improved molecular insights into HCC pathogenesis are critically needed for early detection and targeted intervention, especially as survival remains poor when diagnosed at advanced stages [1,2].

Interleukin-37 (IL-37) is an anti-inflammatory member of the IL-1 cytokine family that broadly suppresses innate and adaptive immune responses [8,9,10,11]. IL-37 can act both intracellularly by binding to Smad3 and influencing gene transcription, and extracellularly via IL-18 receptor α interaction with the co-receptor IL-1R8 (SIGIRR), thereby attenuating pro-inflammatory cytokine production and immune activation [10,11,12]. IL-37 expression is upregulated in inflammatory conditions and has been shown to modulate hepatic inflammation, fibrogenesis, and immune cell activation in experimental liver injury models [11,13]. Serum IL-37 levels correlate with disease severity in cirrhosis in some cohorts, suggesting its potential as a marker of chronic liver damage and inflammatory burden [11,13,14].

Emerging evidence indicates a role for IL-37 in hepatocellular carcinoma biology. Several studies have reported reduced IL-37 expression in HCC tumour tissues compared with adjacent non-tumour liver, with lower IL-37 levels associated with larger tumour size, more aggressive features, and poorer survival outcomes [13,14,15,16]. Functional work demonstrates that IL-37 suppresses tumour cell proliferation and shifts oncogenic signalling pathways towards tumour suppression, for example by promoting G2/M cell cycle arrest and modulating Smad3 phospho-isoform signalling to favour anti-proliferative pathways [13,15]. IL-37 has also been implicated in altering tumour immune infiltrates, including recruitment of natural killer (NK) cells and attenuation of immunosuppressive myeloid populations within the HCC microenvironment, supporting a multifaceted role in tumour regulation [13,15,16].

Currently, AFP is a major biomarker used in HCC, but has limited utility providing rationale for expanded biomarker testing. Despite widespread use in clinical practice, alpha-fetoprotein (AFP) has significant limitations as an HCC biomarker. Its sensitivity and specificity are suboptimal, particularly in patients with advanced cirrhosis and decompensated liver function, where AFP may be elevated due to inflammation or remain normal despite the presence of malignancy [17,18,19,20]. Approximately 30–40% of HCC patients do not exhibit significant AFP elevation, and false positives can occur in benign liver conditions, reducing its utility as a standalone diagnostic tool [19,20]. For this reason, additional biomarkers such as des-γ-carboxy prothrombin (DCP) and AFP-L3 have been proposed, but none have fully overcome these challenges in decompensated cirrhosis; composite models such as GALAD are being explored to improve detection [17,21].

In decompensated cirrhosis, where systemic and intrahepatic inflammation drive both liver failure and carcinogenesis, there is an urgent need for novel biomarkers that can accurately detect HCC and stratify risk independent of AFP’s limitations [17,18,19,21]. Given IL-37’s immunomodulatory properties, correlation with disease severity in cirrhosis, and emerging evidence of links to tumour biology, this study aims to evaluate whether IL-37 may serve as a reliable biomarker for HCC in patients with decompensated chronic liver disease [8,9,10,11,12,13,14,16]. Identifying such biomarkers could enhance surveillance strategies and improve outcomes in a high-risk population with limited diagnostic options [17,18,19,21].

## 2. Materials and Methods

### 2.1. Study Design and Patient Population

This prospective observational study analyzed circulating IL-37 levels in a total of 221 patients with advanced chronic liver disease (ACLD). Among these, 54 patients had a diagnosis of HCC, while 167 patients had ACLD without evidence of HCC. Patients were consecutively recruited during routine clinical care at the hepatology outpatient clinic or during inpatient admissions at the Department of Internal Medicine I (Gastroenterology, Hepatology, Endocrinology and Metabolism), Medical University of Innsbruck. Enrollment occurred between 2018 and 2020. Patients were categorized into four groups based on disease stage and HCC status: cACLD with HCC, cACLD without HCC, dACLD with HCC, and dACLD without HCC.

Serum IL-37 was measured once per participant at the time of clinical assessment, either during an outpatient visit or an inpatient stay. Blood sampling for IL-37 quantification and routine laboratory parameters was performed concurrently. Clinical data were collected at the same time point and included demographic variables, liver disease etiology, disease severity, and the presence of hepatic decompensation at the time of sampling. All patients with HCC were treated with radiofrequency ablation. Patients underwent clinical follow-up over a 5-year period.

Clinical and laboratory data were retrieved and documented using the institutional electronic medical record system. Collected variables included serum IL-37 concentrations (ng/mL), demographic characteristics (e.g., age, sex, body mass index), liver disease etiology, indicators of disease severity (including Model for End-Stage Liver Disease (MELD) score, decompensation status, and Barcelona Clinic Liver Cancer (BCLC) stage), and standard laboratory parameters [18,22,23].

### 2.2. Quantification of Serum IL-37

Serum concentrations of human IL-37 were quantified using a commercially available sandwich enzyme-linked immunosorbent assay (ELISA) (IL-37 Human ELISA Kit, Cat. No. AG-45A-0041YEK-KI01, AdipoGen Life Sciences, Fuellinsdorf, Switzerland) according to the manufacturer’s instructions.

Blood samples were collected by venipuncture, centrifuged at 1200× *g* for 15–20 min, and serum aliquots were stored at ≤−80 °C until batch analysis. Prior to analysis, samples were thawed once and handled uniformly. Calibration standards were prepared by reconstituting the lyophilized IL-37 standard to a concentration of 2 ng/mL, followed by generation of a seven-point, two-fold serial dilution ranging from 1.0 to 0.016 ng/mL in ELISA Buffer 1X supplied with the kit, including a zero standard. One hundred microliters of standards and serum samples were added in duplicate to microtiter plates pre-coated with an anti-human IL-37 polyclonal antibody and incubated overnight at 4 °C. After washing three times with Wash Buffer 1X supplied with the kit, plates were incubated with 100 µL of detection antibody (1:1000 dilution in ELISA Buffer 1X) for 1 h at 37 °C, followed by further washing steps. Subsequently, wells were incubated with horseradish peroxidase (HRP)-conjugated anti-rabbit IgG (1:100 dilution) for 1 h at 37 °C and washed five times. Enzymatic activity was visualized by adding 100 µL of tetramethylbenzidine (TMB) substrate for 10 min at room temperature in the dark. The reaction was stopped with 100 µL of stop solution, and absorbance was measured at 450 nm using a microplate reader within 30 min. IL-37 concentrations were calculated using a quadratic standard curve. The assay range was 0.016–1.0 ng/mL, with a lower limit of detection of 10 pg/mL. Final concentrations were corrected for dilution factors. Samples exceeding the upper measurement range were diluted (1:2 or 1:5) in ELISA buffer and reassessed according to the manufacturer’s recommendations [24].

### 2.3. Clinical Definitions

Hepatic decompensation was defined as the transition from compensated to decompensated ACLD in accordance with the Baveno VII consensus criteria [25]. Patients were classified as decompensated if, at the time of IL-37 sampling, they presented with at least one of the following clinical events: ascites, variceal bleeding, clinically overt hepatic encephalopathy, or jaundice, reflecting clinically relevant deterioration of liver function. All patients categorized as decompensated were experiencing an active decompensating event at the time of assessment.

Clinically overt hepatic encephalopathy (HE) was defined according to the West Haven classification as grade II or higher [26]. This included clearly recognizable neuropsychiatric abnormalities such as disorientation, behavioral changes, asterixis, impaired consciousness, or progression to stupor or coma. Diagnosis was based on clinical documentation, with systematic exclusion of alternative causes of acute encephalopathy, including infection, gastrointestinal bleeding, constipation, electrolyte disturbances, renal dysfunction, and sedative medication use [26].

Alcohol-related liver disease (ALD) was defined as chronic liver disease with relevant alcohol consumption (>50 g/day in women and >60 g/day in men) as the predominant etiological factor, in the absence of a dominant metabolic cause of steatosis. Patients with alcoholic hepatitis were excluded [27]. Metabolic dysfunction–associated alcohol-related liver disease (metALD) was defined by the presence of hepatic steatosis, relevant alcohol intake (>20 g/day in women and >30 g/day in men), and concomitant metabolic dysfunction (e.g., obesity, type 2 diabetes mellitus, arterial hypertension, or dyslipidemia), in line with current nomenclature [28]. Metabolic dysfunction–associated steatotic liver disease (MASLD) was diagnosed according to international consensus criteria, requiring hepatic steatosis in combination with at least one cardiometabolic risk factor, in the absence of relevant alcohol consumption or competing liver disease etiologies [28].

Chronic hepatitis B virus (HBV) infection was defined by detectable serum HBV DNA using quantitative PCR. Chronic hepatitis C virus (HCV) infection was defined by detectable serum HCV RNA by PCR. Hepatitis D virus (HDV) infection was diagnosed in HBV-infected patients by detectable HDV DNA [29]. HCC was diagnosed non-invasively using contrast-enhanced multiphasic computed tomography (CT) or magnetic resonance imaging (MRI), according to EASL criteria. Typical imaging hallmarks included arterial phase hyperenhancement with portal venous and/or delayed phase washout, with or without capsule appearance. Lesions were categorized using LI-RADS, with LI-RADS 5 considered diagnostic for HCC. Tumour stage was assigned using the BCLC staging system [17,23,30].

### 2.4. Statistical Analysis

Continuous variables were summarized as median with first and third quartiles (interquartile range, IQR), while categorical variables are presented as absolute and relative frequencies (Table 1). Normality assumptions were not met, as assessed by visual inspection and distribution characteristics; therefore, non-parametric tests were applied throughout. Clinical characteristics between groups were compared using the Mann–Whitney U test for continuous variables and the χ^2^ test or Fisher’s exact test, as appropriate, for categorical variables (SciPy v1.16.3). Group-wise comparisons of IL-37 and AFP levels were consistently performed using the Mann–Whitney U test.

Correlations between CRP, INR, and AFP levels were assessed using Spearman’s rank correlation coefficient. To account for multiple comparisons, *p*-values were adjusted using the Benjamini–Hochberg procedure to control the false discovery rate (FDR), with adjusted *p*-values reported where appropriate.

For subgroup analyses, patients were stratified according to AFP levels using a predefined cutoff of 20 ng/mL [17,19], and IL-37 levels were dichotomized at the cohort median (50th percentile; 0.1832 ng/mL). Sensitivity analyses were performed to confirm the robustness of results across stratified groups.

Transplant-free survival in patients with HCC, defined as a composite endpoint of death or liver transplantation, was analyzed using the lifelines package (v0.30.0), with administrative censoring at 3 years. Kaplan–Meier survival curves were generated and compared after stratification by IL-37 median levels [31]. In addition, a Cox proportional hazards regression model was fitted with IL-37 as a continuous variable, adjusted for age and sex. Hazard ratios (HRs) with 95% confidence intervals (CIs) were reported, and proportional hazards assumptions were assessed using standard diagnostic methods.

All statistical analyses were conducted in Python (v3.13.11), with data handling performed using Pandas (v2.3.3) and NumPy (v2.3.5). Data visualization was carried out using Matplotlib (v3.10.8) and Seaborn (v0.13.2). All statistical tests were two-sided, and a *p*-value < 0.05 was considered statistically significant.

### 2.5. Ethical Considerations

The study was conducted in accordance with the Declaration of Helsinki and approved by the Ethics Committee of the Medical University of Innsbruck (approval number: AN2017-0016 369/4.21). Written informed consent was obtained from all participants prior to study inclusion.

## 3. Results

### 3.1. Clinical Characteristics of ACLD Patients with and Without HCC

Clinical characteristics of ACLD patients with and without HCC showed notable differences in age and disease severity. Baseline demographics and clinical features of the 221 patients are summarized in Table 1. Patients with HCC were predominantly male and older than those without HCC. Differences in liver disease severity were observed between groups, with HCC patients less frequently presenting with decompensated ACLD and showing lower MELD scores. AFP levels varied between patients with and without HCC, as well as across compensated and decompensated HCC subgroups. Group-specific differences in median serum IL-37 concentrations prompted further stratified analyses according to disease stage (cACLD vs. dACLD), Child–Pugh class, tumour stage, and AFP levels.

### 3.2. IL-37 Is Significantly Reduced in Decompensated ACLD Patients with HCC

**Figure 1:**
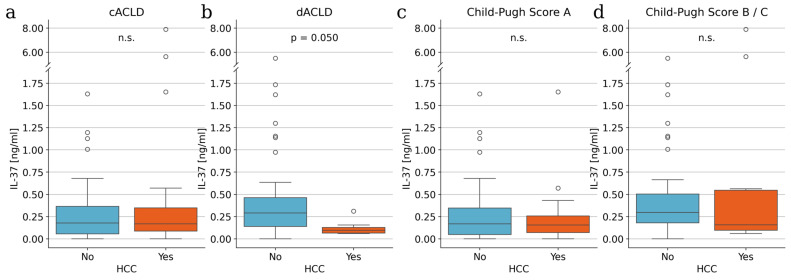
IL-37 levels in patients with and without HCC. IL-37 levels in (**a**) cACLD and (**b**) dACLD patients, and in (**c**) patients with Child-Pugh Score A and (**d**) Child-Pugh Scores B and C. *p*-values were determined using Mann–Whitney U tests. n.s., not significant.

To evaluate whether IL-37 discriminates between patients with and without HCC across different stages of liver disease, serum IL-37 concentrations were analyzed according to ACLD stage and Child–Pugh class. In patients with cACLD, IL-37 levels did not differ significantly between those with and without HCC (Figure 1a). In contrast, among patients with dACLD, IL-37 concentrations were significantly lower in patients with HCC compared with those without HCC (Figure 1b), suggesting particular informativeness in advanced liver disease where traditional biomarkers such as AFP often lose diagnostic accuracy. Stratification by Child–Pugh class revealed a more nuanced pattern. In patients with preserved liver function (Child–Pugh A), IL-37 levels did not differ significantly between patients with and without HCC (Figure 1c). Similarly, no statistically significant differences were observed in patients with Child–Pugh B or C disease (Figure 1d). These findings suggest that while Child–Pugh class alone may not sufficiently capture IL-37–related differences, the presence of clinically overt dACLD identifies a subgroup in which IL-37 is markedly reduced in the presence of HCC.

### 3.3. IL-37 Shows an Inverse Relationship with AFP in cACLD but Not dACLD

**Figure 2:**
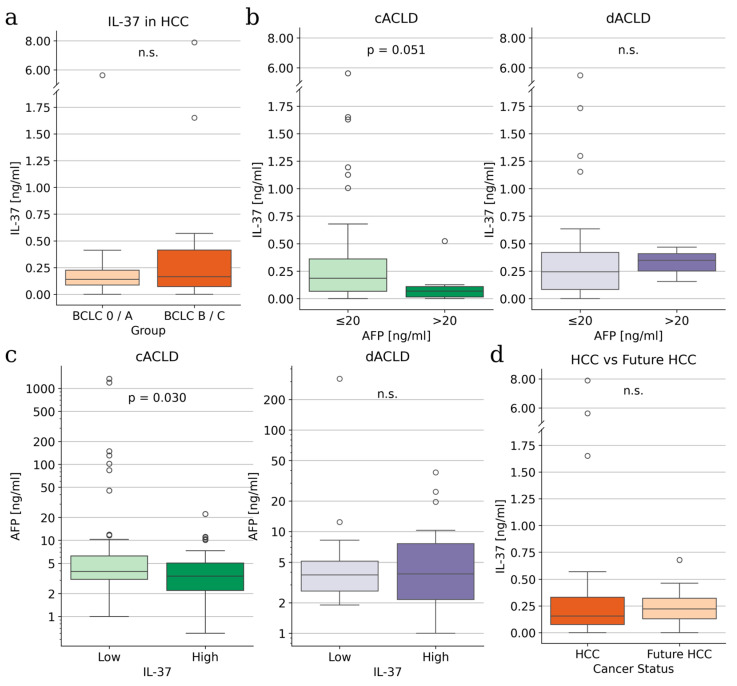
IL-37 and AFP levels in patients with chronic liver disease. (**a**) IL-37 levels in patients with BCLC stages 0 and A compared with stages B and C. (**b**) IL-37 levels in patients with cACLD (green) and dACLD (purple) stratified by low versus high AFP levels. (**c**) AFP levels in patients with low versus high IL-37, defined by the 50th percentile (0.1832 ng/mL), across cACLD and dACLD patients. (**d**) IL-37 levels in patients with existing HCC versus patients who developed HCC during follow-up. *p*-values were determined using Mann–Whitney U tests. n.s., not significant.

Given the limited diagnostic performance of AFP in advanced liver disease, we next explored the relationship between IL-37, AFP, and tumour stage. IL-37 levels did not differ significantly between patients with early-stage HCC (BCLC 0/A) and those with intermediate or advanced disease (BCLC B/C) (Figure 2a), indicating that IL-37 concentrations are not primarily driven by tumour burden. When stratifying patients by AFP levels, a complementary and stage-dependent pattern emerged. In patients with cACLD, IL-37 concentrations were significantly lower in those with elevated AFP compared with patients with low AFP levels (Figure 2b). In contrast, this association was not observed in patients with dACLD. Conversely, AFP levels were significantly higher in patients with low IL-37 concentrations (defined by the cohort median of 0.1832 ng/mL), again predominantly in the cACLD subgroup (Figure 2c). These inverse associations suggest that IL-37 and AFP may capture overlapping but not identical biological information in compensated disease, where AFP retains some discriminatory capacity. In decompensated ACLD, AFP levels were inversely associated with CRP (r=−0.38, $p_{adj}=0.021$) and positively associated with INR (r=0.33, $p_{adj}=0.025$), suggesting that AFP may be influenced by systemic inflammation and liver dysfunction, potentially reducing its specificity for tumour burden. IL-37 levels were additionally compared between patients with HCC at baseline and those who developed HCC during follow-up. Although this comparison did not reach statistical significance, patients with prevalent HCC exhibited numerically lower IL-37 concentrations than patients who subsequently developed HCC (Figure 2d), supporting the hypothesis that declining IL-37 levels may accompany tumour manifestation and potentially precede clinically overt HCC.

### 3.4. IL-37 May Be Associated with Transplant-Free Survival in Patients with HCC, Transplant-Free Survival, with Death or Liver Transplantation

**Figure 3:**
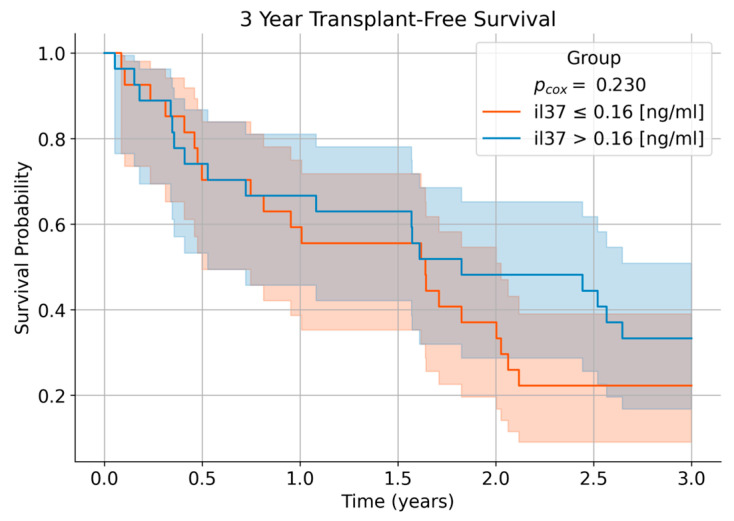
3-year transplant-free survival of HCC patients. For Kaplan–Meier analysis, patients were stratified by low versus high IL-37 levels, defined by the 50th percentile. The *p*-value was determined using a Cox proportional hazards model with IL-37 as a continuous covariate, adjusting for age and sex, with both death and liver transplantation considered as events.

Counted as events, in patients with HCC was analyzed using Kaplan–Meier estimates, stratifying patients by low versus high IL-37 concentrations based on the median value (Figure 3). Patients with higher IL-37 levels demonstrated a trend toward improved transplant-free survival compared with those with lower IL-37 levels, with incidence rates of 41 versus 46 events per 100 person-years, respectively. Cox proportional hazards modeling incorporating IL-37 as a continuous covariate and adjusting for age and sex yielded a hazard ratio of 1.14 (95% CI 0.92–1.41; *p* = 0.23), indicating a consistent but non-significant association between IL-37 levels and survival. Although this analysis was not powered to detect small survival differences, the directionality of the effect suggests a potential prognostic relevance of IL-37. Collectively, the subgroup-specific associations, inverse relationship with AFP, and observed trends across diagnostic and survival analyses align with the concept that IL-37 reflects biologically relevant pathways related to tumour presence and host response.

## 4. Discussion

In this prospective cohort of patients with ACLD, we investigated circulating IL-37 in the context of HCC and examined its relationship with AFP, liver disease severity, and clinical outcome. Our results indicate that IL-37 levels are significantly reduced in patients with HCC in the setting of dACLD and exhibit a distinct pattern of association with AFP in cACLD. Furthermore, IL-37 demonstrated trends consistent with tumour manifestation and survival outcomes, supporting its potential role as a complementary biomarker in advanced liver disease [11,13,14,15,16,19,20,21].

IL-37, an anti-inflammatory cytokine, has gained considerable interest for its immunoregulatory and anti-tumour properties [8,9,10,11,12]. Preclinical studies have shown that IL-37 suppresses hepatocellular carcinoma growth and modulates key signaling pathways implicated in tumour progression. For example, IL-37 can convert oncogenic JNK/pSmad3L/c-Myc signaling to tumour-suppressive pSmad3C/p21 signaling in HCC models, indicative of a direct tumour inhibitory effect; IL-37 has also been reported to induce autophagy and apoptosis in HCC cells via inhibition of the PI3K/AKT/mTOR pathway [13,14,15,16]. Clinical studies corroborate these mechanistic insights: reduced IL-37 expression has been documented in HCC tissues compared with adjacent non-tumour liver, with low expression correlating inversely with serum AFP levels and with poorer outcomes [13,14,15,16]. Additionally, IL-37 may modulate the tumour immune microenvironment by enhancing recruitment of NK cells and altering macrophage polarization toward anti-tumour phenotypes [13,16]. Our findings of lower circulating IL-37 in dACLD patients with HCC extend these molecular data into a clinical context in which immune dysregulation and inflammation underpin disease progression [3,4,5,6,7,11]. Reduced IL-37 levels in patients with dACLD and HCC may reflect an impaired anti-inflammatory and immunoregulatory response, potentially contributing to a pro-tumourigenic microenvironment characterized by increased systemic inflammation and immune dysfunction. In addition, decreased IL-37 may be associated with advanced liver disease severity and immune exhaustion, thereby limiting its protective effects on both innate and adaptive immune pathways in HCC development [8,32,33]. This is particularly relevant as inflammatory cytokines play established roles in liver disease pathogenesis and progression, including cirrhosis and HCC development [3,4,5,6,7,14,34].

AFP remains widely used for HCC surveillance but has limited sensitivity and specificity, particularly in advanced liver disease where levels may be elevated without malignancy or normal despite cancer [17,18,19,20]. In our cohort, although AFP levels differed significantly between patients with and without HCC, its overall diagnostic performance remained limited in the cirrhotic setting. Notably, a considerable proportion of patients with HCC, particularly those with advanced liver disease, exhibited normal or low AFP concentrations, highlighting the limited diagnostic utility of AFP in this setting [19,20]. Importantly, IL-37’s pattern of association with AFP was context-dependent: in cACLD, IL-37 was inversely correlated with AFP, whereas in dACLD this relationship was absent. This suggests that IL-37 and AFP may reflect different aspects of disease biology. AFP is more closely linked to tumour antigen expression, and IL-37 to immune regulation and potentially tumour-associated inflammation [8,9,10,11,12,17,18,19,20]. In advanced liver disease, systemic inflammation and hepatic dysfunction can confound AFP levels, reducing its diagnostic value, while IL-37, an immunomodulatory cytokine, may be less susceptible to such confounding and thus provide complementary information, particularly in dACLD where AFP performance is poorest [11,13,14,15,17,18,19,20]. The inverse relationship between IL-37 and AFP in cACLD may reflect early immune responses to tumourigenesis, where declining IL-37 coincides with rising AFP as malignant clones emerge [13,14,15,16,19,20]. These findings align with evidence indicating that intrahepatic IL-37 is suppressed in HCC and negatively correlated with AFP, suggesting that IL-37 downregulation may be integral to tumour biology rather than a mere epiphenomenon [13,14,15,16].

While our survival analysis did not achieve statistical significance, patients with higher IL-37 levels exhibited a trend toward improved transplant-free survival, consistent with data demonstrating that high IL-37 expression in tumour tissues correlates with better prognosis [13,14,15,16]. The non-significant *p*-value may reflect limited power and underscores the need for larger cohorts to validate prognostic associations. Nonetheless, the directionality aligns with the biological role of IL-37 as an anti-inflammatory and potential anti-tumour mediator [8,9,10,11,12,13,14,15,16]. Furthermore, the observation that patients with prevalent HCC had numerically lower IL-37 than those who developed HCC later suggests that IL-37 decline may precede clinical tumour manifestation. This temporal pattern raises the possibility that IL-37 could serve as an early indicator of malignant transformation in cirrhosis, though longitudinal studies with serial IL-37 measurements are necessary to test this hypothesis [13,14,15,16].

The identification of reliable biomarkers for HCC in dACLD remains an unmet clinical need, as current surveillance modalities, including ultrasound and AFP, lack sufficient sensitivity and specificity in this vulnerable population [17,18,19,20,21]. IL-37’s association with HCC in dACLD patients highlights its potential utility either as a standalone marker or as part of a multi-analyte panel that captures both tumour and host immune dynamics. Measurement of IL-37 may be especially valuable in stratifying risk among patients with dACLD, informing surveillance strategies and potentially identifying candidates for early intervention [17,18,19,20,21]. Additionally, combining IL-37 with existing biomarkers (e.g., DCP, AFP-L3) and clinical parameters could improve predictive performance and offer a more comprehensive biomarker framework for HCC detection in advanced liver disease [17,19,20,21].

This study has several limitations that should be considered when interpreting the findings. First, the relatively small sample size of the HCC subgroup may have limited statistical power for certain stratified analyses and survival estimates, necessitating cautious interpretation of non-significant trends. Accordingly, our findings require confirmation in larger, independent cohorts. Due to the limited sample size, liver function parameters and tumour stage were not included in the survival analysis. Larger multicenter cohorts will be essential for validation. IL-37 was measured at a single time point, precluding assessment of temporal dynamics in relation to disease evolution or response to therapy. Serial measurements could provide valuable insight into whether IL-37 trajectories anticipate HCC development or correlate with clinical events. Accordingly, future studies should incorporate longitudinal measurements alongside tissue-based and histological IL-37 measurements to further elucidate the role of IL-37. While we accounted for major clinical variables, residual confounding by unmeasured factors, including concomitant treatments, comorbidities, and other immune markers, may influence IL-37 levels. Incorporating comprehensive immune profiling would help elucidate the interplay between IL-37 and broader inflammatory networks [11,13,14,15]. The study did not assess tissue expression of IL-37 or its relationship with intrahepatic immune infiltrates. Correlating serum levels with tissue expression and immune contexture could refine mechanistic understanding and strengthen biomarker rationale [13,14,15,16]. Finally, assay variability and pre-analytical factors inherent to cytokine measurement may introduce noise, and cross-platform validation would enhance confidence in the generalizability of IL-37 quantification [24].

Future research should focus on validating IL-37 in larger, independent cohorts with longitudinal follow-up to determine predictive performance and temporal patterns. Integration of IL-37 into multi-marker panels, potentially alongside AFP, DCP, and novel immune biomarkers, could enhance diagnostic accuracy. Mechanistic studies examining IL-37’s interplay with tumour and immune cells in cirrhotic livers will further clarify its role in hepatocarcinogenesis and may reveal therapeutic implications [11,13,14,15,16,19,20,21].

## 5. Conclusions

In conclusion, circulating IL-37 is significantly reduced in patients with HCC in the context of decompensated ACLD and shows a complementary pattern of association with AFP in compensated disease. These findings support IL-37 as a promising biomarker candidate that reflects immune and inflammatory dynamics pertinent to HCC and advanced liver disease. Although further validation is required, IL-37 has the potential to improve risk stratification and early detection strategies for HCC, particularly in patients with decompensated cirrhosis where conventional biomarkers are limited [11,13,14,15,16,19,20,21].

## Figures and Tables

**Table 1:** Demographics and disease characteristics of patient cohorts.

Characteristic	Overall *n* = 221	Cirrhosis Without HCC *n* = 167	Cirrhosis with HCC *n* = 54	*p*-Value
**Sex**				0.005
Male	162 (73%)	114 (68%)	48 (89%)	
Female	59 (27%)	53 (32%)	6 (11%)	
**Age**	69 (64, 77)	67 (63, 75)	75 (69, 80)	<0.001
**BMI**				0.69
Normal	70 (32%)	59 (30%)	15 (28%)	
Overweight	66 (30%)	55 (33%)	16 (30%)	
Obese	42 (19%)	30 (18%)	12 (22%)	
**Etiology**				0.55
Genetic	6 (2.3%)	6 (3.6%)	0 (0%)	
Immune mediated	14 (6.3%)	11 (6.6%)	3 (5.6%)	
SLD	149 (67%)	111 (67%)	38 (70%)	0.036
ALD	68 (31%)	58 (35%)	10 (19%)	
MASH	1 (0.5%)	0 (0%)	1 (1.9%)	
MASLD	44 (20%)	29 (17%)	15 (28%)	
MetALD	45 (20%)	33 (20%)	12 (22%)	
Viral	52 (24%)	39 (23%)	13 (24%)	0.70
Hepatitis B	13 (5.9%)	10 (6.0%)	3 (5.6%)	
Hepatitis C	37 (17%)	28 (17%)	9 (17%)	
Hepatitis D	2 (0.9%)	1 (0.6%)	1 (1.9%)	
**MELD**	10 (8, 13)	10 (8, 13)	9 (7, 11)	0.053
**Child–Pugh Score**				0.070
A	149 (67%)	109 (65%)	40 (74%)	
B	57 (26%)	43 (26%)	14 (26%)	
C	15 (6.8%)	15 (9.0%)	0 (0%)	
**ACLD**				0.017
cACLD	163 (74%)	116 (70%)	47 (87%)	
dACLD	58 (26%)	51 (30%)	7 (13%)	
**AFP**	3.80 (2.40, 5.70)	3.50 (2.30, 4.98)	5.90 (3.65, 10.6)	<0.001
**IL-37**	0.20 (0.07, 0.38)	0.24 (0.07, 0.38)	0.16 (0.08, 0.33)	0.27
**Characteristic**	**Overall** *n* = 54	**cACLD with HCC** *n* = 10	**dACLD with HCC** *n* = 7	***p*** **-value**
**Age**	75 (69, 80)	76 (69, 81)	72 (66, 77)	0.21
**BCLC**				1.0
0/A	28 (52%)	24 (51%)	4 (57%)	
B/C	26 (48%)	23 (49%)	3 (43%)	
**AFP**	3.80 (2.40, 5.70)	5.45 (3.50, 10.3)	8.20 (8.00, 12.4)	0.20
**Characteristic**	**Overall** *n* = 64	**HCC during follow-up** *n* = 10	**Prevalent HCC** *n* = 54	***p*** **-value**
**Age**	74 (68, 79)	69 (64, 73)	75 (69, 80)	0.09
**IL-37**	0.16 (0.08, 0.33)	0.22 (0.13, 0.32)	0.16 (0.08, 0.33)	0.51

## Data Availability

The data that support the findings of this study are available from the corresponding author upon reasonable request. Due to ethical and institutional restrictions, the raw data are not publicly available.
